# Maintaining Life Satisfaction in Adolescence: Affective Mediators of the Influence of Perceived Emotional Intelligence on Overall Life Satisfaction Judgments in a Two-Year Longitudinal Study

**DOI:** 10.3389/fpsyg.2015.01892

**Published:** 2015-12-22

**Authors:** Nicolás Sánchez-Álvarez, Natalio Extremera, Pablo Fernández-Berrocal

**Affiliations:** ^1^Emotions Lab, Faculty of Psychology, University of MálagaMálaga, Spain; ^2^Department of Social Psychology, Faculty of Psychology, University of MálagaMálaga, Spain; ^3^Department of Basic Psychology, Faculty of Psychology, University of MálagaMálaga, Spain

**Keywords:** perceived emotional intelligence, subjective well-being, life satisfaction, positive and negative affect, mediation, positive psychology

## Abstract

Much attention has been paid to the psychological processes underlying the improvement in mood states and human well-being, particularly during adolescence. Theoretical and empirical research suggests that emotional skills may play a role in enhancing perceived well-being; however, the mechanisms involved in during adolescence are unclear. The purpose of this study was to extend understanding by investigating the potential mediators of the relationship between emotional intelligence (EI) and life satisfaction in a 2-years study. Participants were 269 high school students (145 girls and 124 boys) who completed the self-report perceived emotional intelligence (PEI) Scale, the Satisfaction with Life Scale, and the Positive Affect and Negative Affect Scale three times at 1-year intervals. The three-step longitudinal design corroborated earlier research indicating that positive and negative affect mediate the relationships between EI and life satisfaction. Students with high PEI tended to have more positive experiences and fewer negative experiences, which contributed to their greater life satisfaction. No sex differences were found in the multi-group analyses, suggesting that the causal relationships are similar in both sexes. These findings extend our understanding of the complex network of relationships involving PEI and life satisfaction in adolescence. Implications and limitations of the findings are discussed.

## Introduction

In recent decades many studies have reported positive relationships between emotional skills and well-being outcomes (Mikolajczak et al., 2008; Zeidner et al., 2009). Although there is growing interest in identifying the psychological processes that enhance positive emotions and life satisfaction, there is a lack of understanding of the emotional processes which underpin subjective well-being. Research indicates that emotional skills play a vital role in coping with stress (Salovey et al., 1999), suggesting that inappropriate use of emotional skills might result in various emotional disorders (Gohm and Clore, 2002a; Nolen-Hoeksema, 2003). There is, however, a dearth of scientific literature on the relationship between emotional skills and well-being (Sánchez-Álvarez et al., in press). This study focuses on the influence of emotional self-efficacy, also known as perceived emotional intelligence (PEI), and subjective well-being (SWB), and considers how the different components of SWB (cognitive and affective) might act as mediators.

### PEI and Cognitive SWB

The concept of PEI should be defined relative to trait emotional intelligence (EI) and ability EI (Petrides and Furnham, 2000). Following the development of the theory of EI (Mayer and Salovey, 1997) two approaches to evaluating emotional skills have emerged: self-assessment scales such as the Trait Meta-Mood Scale (TMMS; Salovey et al., 1995) and ability measures such as the Mayer–Salovey–Caruso Emotional Intelligence Test (MSCEIT 2.0; Mayer et al., 2002), a multi-dimensional measure based on performance on various tasks. Although ability measures are beginning to be recommended (Mayer and Salovey, 1997), self-assessment measures are more commonly used in the mental health field owing to the interest in emotional beliefs (Fernández-Berrocal and Extremera, 2008). Scores on the TMMS, which evaluates an individual’s perception of his or her emotional skills, or PEI (Salovey et al., 2002) have been shown to correlate with many mental health and psychological adjustment variables in non-clinical populations. PEI is a constellation of emotional self-perceptions and thus located at the lower end of Eysenckian and Big Five factor personality hierarchies (Petrides et al., 2007). PEI, as measured with the TMMS, is defined as a continuous, reflective process associated with beliefs about the attention one pays to one’s emotional state (attention to feelings), mood clarity and perceived ability to regulate one’s emotional state (emotional repair) (Salovey and Mayer, 1990). Cognitive SWB is a measure of one’s evaluation of emotional information throughout one’s life (Pavot and Diener, 2008) and is usually evaluated using the Satisfaction with Life Scale (SWLS; Diener et al., 1985). There is some controversy about the nature of the relationship between PEI dimensions and life satisfaction; some studies have reported that life satisfaction was related to attention to feelings, mood clarity and emotional repair (Bastian et al., 2005) whereas others found that only mood clarity and emotional repair were related to life satisfaction (Extremera and Fernández-Berrocal, 2005; Augusto-Landa et al., 2006; Rey et al., 2011), or that only mood clarity (Palmer et al., 2002) or emotional repair was related to life satisfaction (Thompson et al., 2007). In summary, these studies indicate that TMMS-based measures of various PEI dimensions are important predictors of scores on indices of cognitive SWB.

In general, individuals with high perceived ability to interpret emotional information and deal more effectively with emotional issues achieve a greater sense of life satisfaction (Lazarus, 1991; Mayer and Salovey, 1997; Salovey et al., 1999). Although there are several reports that PEI is associated with life satisfaction (Gohm and Clore, 2002b; Palomera and Brackett, 2006; Kong et al., 2012), few longitudinal studies have been carried out (for an exception see Extremera et al., 2011). Experimental and longitudinal research is required to elucidate the causal structure of the relationships between PEI dimensions and life satisfaction.

### PEI and Affective SWB

Affective well-being represents the balance between an individual’s experience of positive and negative emotions (Diener et al., 1999) and is usually evaluated using the Positive Affect and Negative Affect Scale (PANAS; Watson et al., 1988). Certain PEI dimensions are associated with high rates of positive emotions and lower rates of negative emotions (Palmer et al., 2002; Palomera and Brackett, 2006; Gallagher and Vella-Brodrick, 2008; Augusto-Landa et al., 2012; Koydemir and Schütz, 2012). Attention to feelings is positively correlated with negative affect (Augusto-Landa et al., 2006; Thompson et al., 2011), and negatively correlated with positive affect (Palmer et al., 2002; Augusto-Landa et al., 2006; Palomera and Brackett, 2006) whereas mood clarity is negatively correlated with negative affect (Palmer et al., 2002; Palomera and Brackett, 2006) and positively correlated with positive affect (Gohm and Clore, 2002a; Palmer et al., 2002; Augusto-Landa et al., 2006). Like mood clarity, emotional repair is negatively correlated with negative affect (Augusto-Landa et al., 2006; Palomera and Brackett, 2006) and positively with positive affect (Gohm and Clore, 2002a; Kafetsios and Zampetakis, 2008; Augusto-Landa et al., 2012). This body of research suggests that individuals with high PEI may spend less time in negative moods and more time in positive moods relative to those with low PEI (Salovey and Mayer, 1990; Koydemir and Schütz, 2012). Again, most of this empirical evidence is from cross-sectional studies, although there has been one longitudinal study (Ciarrochi et al., 2008).

### PEI, Cognitive, and Affective SWB

The two components of SWB are considered separate constructs although they are moderately associated (Diener, 1994). PEI is differently related to the two components, offering evidence of continuity of the processes involved in SWB (Brackett and Mayer, 2003; Austin et al., 2005; Schutte and Malouff, 2011). Some research has suggested that PEI, positive and negative affect and life satisfaction are sequentially related (Karademas, 2007; Vergara et al., 2015). Affective SWB is a measure of one’s current experience of positive and negative emotions, whereas cognitive SWB is one’s assessment of emotions experienced in the past and present (Diener, 1994). This implies the existence of a direct path from affective SWB to cognitive SWB (Augusto-Landa et al., 2006; Kesebir and Diener, 2008); in other words, individuals rely on the balance between their experiences of pleasure and displeasure to judge their life satisfaction (Schimmack et al., 2008). Schimmack et al. (2002) found that affective SWB was directly influenced by certain personality traits, whilst their influence on cognitive SWB was mediated by affective SWB. Another psychological process which might underlie this relationship is the ability to use the information provided by emotions intelligently, which is essential to physical and psychological adaptation (Mayer and Salovey, 1997). Gignac (2006) and Kong and Zhao (2013) devised and tested models of the relationship between PEI and life satisfaction in young adults, demonstrating that it is mediated by certain affective processes. Both studies used structural equation modeling with latent variables based on a global PEI variable and therefore did not provide information about the relationships in which specific aspects of PEI were involved. The lack of findings in previous studies, where the relationship between the different dimensions of PEI with affective processing and life satisfaction, is not described (Kong and Zhao, 2013). Previous research indicated that attention to the feelings was prospectively related to negative affect, but not positive affect (Thompson et al., 2011). There is also some evidence that there positive emotions are strongly associated with mood clarity and emotional repair (Palmer et al., 2002; Palomera and Brackett, 2006). As the various TMMS subscores relate to different aspects of emotional skill their relationships with the two aspects of SWB may also vary.

Because all the studies discussed above were cross-sectional they do not provide evidence about causality (Gignac, 2006; Kong and Zhao, 2013). Longitudinal data are needed to determine the direction of causal relationships (Willett and Sayer, 1996). In an emerging field such as SWB research, longitudinal studies have the potential to provide evidence that would otherwise remain lacking. For example, Wright (2007) pointed out that one of the advantages of longitudinal research is that it allows the researcher to model time as an independent variable. Whereas theories often explicitly state the importance of time, longitudinal data actually allow the use of time as a research variable. Second, in a meta-analysis (Conley, 1984) it was pointed out that longitudinal data enable one to estimate the temporal stability of a construct, i.e., determine the extent of intra-individual temporal variance. Cross-sectional research investigates inter-individual variance in order to determine associations between variables at individual level, whereas longitudinal data also provide information about intra-individual variance over time. Longitudinal data can address questions about temporal fluctuation in scores (do individuals’ responses vary significantly over time) or questions related to both intra- and inter-individual variance (e.g., do attitudes fluctuate more over time in neurotics than emotionally stable individuals?). Longitudinal research can also yield information about the sequence of changes in variables or responses. If we can show, for example, that an increase in PEI is followed by an increase in SWB and that a decrease in PEI is followed by a decrease in SWB this provides some basis for inferring a causal relationship between SWB and PEI, whereas cross-sectional data only provide evidence of an association between the two variables. Although there is no substitute for experimental research when it comes to providing evidence of causality, longitudinal design research can help to tease out complex relationships among variables by asking questions such as, does cognition go before emotion, vice versa, or both? Data on the pattern of temporal changes in a set of variables may lead to insights that cross-sectional research would not. In summary, longitudinal research has several advantages to over cross-sectional research or even experimental research (Avey et al., 2008).

### This Study

This study had three aims. The first was to examine the relationships between the various aspects of PEI, positive and negative affect and life satisfaction. Second, we wanted to use longitudinal research to determine the extent to which the various aspects of PEI could account for life satisfaction after taking into account the role of positive and negative affect. Third, we sought to extend the previous literature by investigating positive and negative affect as potential mediators of the relationship between PEI and life satisfaction over a 2-years period.

Based on earlier findings we expected that PEI, mood clarity and emotional repair would be positively associated with life satisfaction and positive affect, and that attention to feelings would be negatively associated life satisfaction and positively associated with negative affect. We also expected that positive affect would be positively associated with life satisfaction and negatively associated with negative affect. Furthermore, we hypothesized that positive and negative affect would mediate the relationship between PEI and life satisfaction during a 2-years follow-up study.

## Materials and Methods

### Participants

The sample for this study consisted of 269 adolescents from southern Spain (145 girls and 124 boys) recruited from various high schools; they participated voluntarily and anonymously. Participants completed the package of questionnaires at the beginning of the first academic semester in three successive years. The package of questionnaires comprised the TMMS, the PANAS and the SWLS as part of a larger battery of questionnaires used for other purposes. At the first assessment ages ranged from 12 to 16 years (*M* = 13.26, *SD* = 1.05), the mean age of the boys was 13.24 years (*SD* = 1.11) and the mean age of the girls was 13.28 years (*SD* = 1.10). This study was carried out in accordance with the Declaration of Helsinki and ethical guidelines and was approved by the Research Ethics Committee of the University of Málaga.

### Measures

#### Perceived Emotional Intelligence

It was measured with the self-report TMMS (Salovey et al., 1995), which is considered a proxy for PEI (Salovey et al., 2002). The TMMS evaluates the extent to which people attend to and value their feelings (attention to feelings), feel clear rather than confused about their feelings (mood clarity), and use positive thinking to repair negative moods (emotional repair). The shortened Spanish version (Fernández-Berrocal et al., 2004) includes 24 items from the original version (eight for each subscale). The original 48 items were subjected to principal components analysis with varimax rotation. Items with loadings ≤0.40 were removed. The analysis indicated that the scale had three main factors - attention, clarity, and repair – which is consistent with evidence an analysis of the structure of the original English version (Salovey et al., 1995). This Spanish version has shown acceptable internal consistency and satisfactory test–retest reliability and scores are negatively correlated with depression and ruminative responses and positively correlated with life satisfaction. Further details on the scoring, reliability, and validity of the Spanish version of TMMS can be found elsewhere (Fernández-Berrocal et al., 2004).

#### Life Satisfaction

It was measured with the Spanish version of the SWLS (Diener et al., 1985) to assess perceived global life satisfaction. Both English and Spanish versions are considered to have adequate discriminant validity and internal consistency (Diener et al., 1985; Atienza et al., 2003). The SWLS consists of five items to which responses are given on a seven-point Likert scale ranging from 1 = strongly disagree to 7 = strongly agree.

#### Positive Affect and Negative Affect

It was assessed with PANAS (Watson et al., 1988), a twenty-item inventory consisting of 10 adjectives describing positive affect (e.g., excited, interested) and 10 adjectives describing negative affect (e.g., distressed, irritable). The scale was administered in the general format with the instructions to ‘rate the extent to which you generally feel this way’ on a scale ranging from 1 (very slightly or not at all) to 5 (extremely).

### Procedures

The questionnaires were administered to the sample in the following order: TMMS, PANAS, and finally the SWLS. The questionnaires were administered in scheduled high school classes by trained instructors. The average time taken to complete the set of questionnaires was 40 min. A researcher was present throughout testing and participants were encouraged to ask questions about the questionnaires. Informed consent was obtained from all participants.

### Design

We used a three-step, longitudinal design with 1 year between assessments. Our longitudinal model also included the effect of positive affect and negative affect (mediating variables) on the relationship between the TMMS dimensions (independent variables) and life satisfaction (dependent variable).

### Analysis Strategy

We used structural equation modeling (SEM) with maximum likelihood estimation (a common SEM method; Schermelleh-Engel et al., 2003), implemented in the AMOS 20 software, to assess the direct and indirect relationships between investigated variables. The estimated model consisted of three assessments at 1-year intervals. SEM is used to determine predictive relationships between variables (Cole and Maxwell, 2003). The adjusted model was evaluated with residual mean squared error approximation (RMSEA; values <0.08 indicate acceptable fit), normed fix index (NFI), incremental fix index (IFI), and comparative fit index (CFI); for all these fit indices values >0.90 indicate acceptable fit (Kline, 1998).

## Results

### Descriptive Statistics

Means, standard deviations, and pairwise correlations for all variables are presented in **Table 1.** Attention to feelings was positively correlated with negative affect at all three timepoints, whereas mood clarity and emotional repair were positively correlated with positive affect and life satisfaction at all three timepoints.

**Table 1:** Pearson product moment correlation coefficients, means, and coefficient Alphas.

	1	2	3	4	5	6	7	8	9	10	11	12	13	14	15	16	17	18
(1) Attention (T1)	−																	
(2) Clarity (T1)	0.39ˆ**	−																
(3) Repair (T1)	0.25ˆ**	0.54ˆ**	−															
(4) Positive affect (T1)	0.12ˆ**	0.42ˆ**	0.44ˆ**	−														
(5) Negative affect (T1)	0.29ˆ**	−0.01	0.01	−0.10	−													
(6) Life satisfaction (T1)	−0.07	0.24ˆ**	0.29ˆ**	0.46ˆ**	−0.34ˆ**	−												
(7) Attention (T2)	0.46ˆ**	0.08	0.01	0.05	0.14ˆ*	0.01	−											
(8) Clarity (T2)	0.05	0.33ˆ**	0.34ˆ**	0.23ˆ**	−0.16ˆ*	0.17ˆ**	0.17ˆ**	−										
(9) Repair (T2)	0.02	0.16ˆ**	0.42ˆ**	0.22ˆ**	−0.17ˆ**	0.22ˆ**	0.07	0.49ˆ**	−									
(10) Positive affect (T2)	0.02	0.17ˆ**	0.18ˆ**	0.49ˆ**	−0.07	0.35ˆ**	0.04	0.27ˆ**	0.27ˆ**	−								
(11) Negative affect (T2)	0.23ˆ**	0.01	−0.01	−0.08	0.44ˆ**	−0.30ˆ**	0.23ˆ**	−0.05	−0.18ˆ**	0.01	−							
(12) Life satisfaction (T2)	−0.14*	0.12ˆ*	0.17ˆ**	0.29ˆ**	−0.29ˆ**	0.57ˆ**	−0.05	0.24ˆ**	0.31ˆ**	0.43ˆ**	−0.33ˆ**	−						
(13) Attention (T3)	0.38ˆ**	0.19ˆ**	0.14ˆ*	0.13ˆ*	0.13ˆ*	0.09	0.47ˆ**	−0.01	0.07	−0.02	0.02	−0.01	−					
(14) Clarity (T3)	0.15ˆ**	0.34ˆ**	0.27ˆ**	0.27ˆ**	−0.04	0.28ˆ**	0.21ˆ**	0.41ˆ**	0.29ˆ**	0.23ˆ**	−0.08	0.27ˆ**	0.42ˆ**	−				
(15) Repair (T3)	0.10	0.22ˆ**	0.43ˆ**	0.25ˆ**	−0.03	0.22ˆ**	0.06	0.33ˆ**	0.48ˆ**	0.26ˆ**	−0.17ˆ**	0.31ˆ**	0.30ˆ**	0.55ˆ**	−			
(16) Positive affect (T3)	0.02	0.20ˆ**	0.22ˆ**	0.39ˆ**	−0.06	0.28ˆ**	0.01	0.27ˆ**	0.22ˆ**	0.47ˆ**	−0.07	0.28ˆ**	0.19ˆ**	0.45ˆ**	0.39ˆ**	−		
(17) Negative affect (T3)	0.22ˆ**	−0.01	−0.04	−0.05	0.29ˆ**	−0.23ˆ**	0.14ˆ*	−0.01	−0.05	−0.01	0.50ˆ**	−0.22ˆ**	0.19ˆ**	−0.02	−0.08	0.02	−	
(18) Life satisfaction (T3)	−0.10	0.13ˆ*	0.23ˆ**	0.30ˆ**	−0.24ˆ**	0.57ˆ**	−0.14	0.17ˆ**	0.25ˆ**	0.34ˆ**	−0.26ˆ**	0.54ˆ**	0.08	0.34ˆ**	0.34ˆ**	0.36ˆ**	−0.31ˆ**	−
*M*	3.05	3.07	3.35	3.44	2.26	4.82	2.98	3.09	3.30	3.47	2.24	4.90	3.11	3.21	3.32	3.40	2.35	4.90
*SD*	0.81	0.77	0.81	0.69	0.64	1.41	0.79	0.71	0.79	0.65	0.64	1.27	0.79	0.78	0.80	0.58	0.64	1.25
Alpha	0.83	0.79	0.78	0.81	0.79	0.84	0.85	0.81	0.80	0.81	0.80	0.85	0.86	0.86	0.84	0.77	0.79	0.87

*N* = 269; ^∗^*p* < 0.05; ^∗∗^*p* < 0.01.

### Structural Model

**FIGURE 1:**
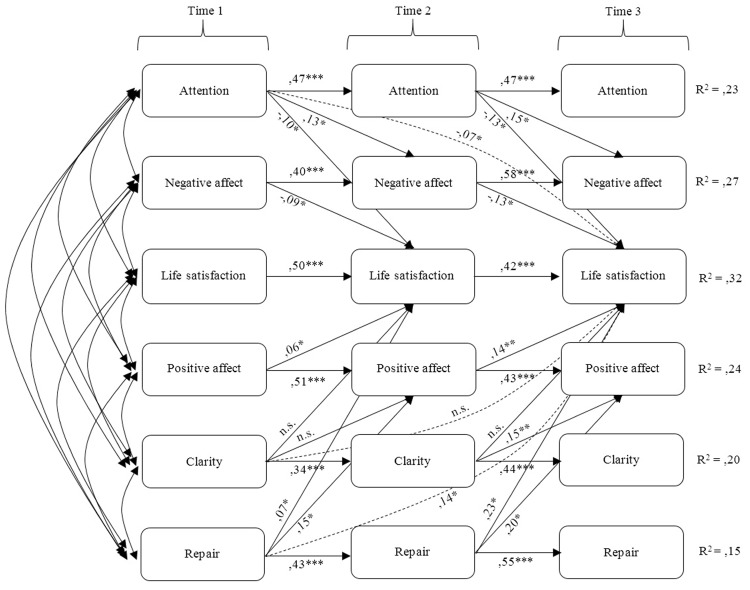
**Three-wave longitudinal model covering the same period of time.** Standardized solution. *N*=269; ^∗^*p* < 0.05; ^∗∗^*p* < 0.01; ^∗∗∗^*p* < 0.001.

Based on earlier research (Gignac, 2006; Kong and Zhao, 2013), we developed a cross-lagged panel model, drawing on mediation models proposed by Baron and Kenny (1986). The model included all measured variables, direct paths from TMMS dimensions to positive affect, negative affect, and life satisfaction, and direct paths from positive affect and negative affect to life satisfaction. Preliminary analysis indicated two types of mediation, one involving positive affect and the other involving negative affect. In accordance with the recommended protocol for the AMOS software we then tested a three-step longitudinal model with two-way mediation. The results are reported in **Figure 1**. Both mediation effects (affection negative way, and positive affect way) are integrated into a single model. This model was an acceptable fit to the data (*X*^2^ = 173,074; *p* = 1.502; *g.l*. = 67; *NFI* = 0.908; *IFI* = 0.941; *CFI* = 0.938; *RMSEA* = 0.073), overall the model accounted for 32% of the variance in life satisfaction.

As shown in **Table 2** there were indirect associations between TMMS dimensions and positive and negative affect and life satisfaction. The strongest indirect associations were those involving attention to feelings and mood clarity; those involving emotional repair were weaker.

**Table 2:** Standardized indirect effects and 95% confidence intervals.

	Attention (T1)	Clarity (T1)	Repair (T1)
Positive affect (T3)	–	0.034	0.012
Negative affect (T3)	0.084	–	–
Life satisfaction (T3)	−0.026	0.027	0.016

To check the robustness of the three-step longitudinal model, the integrity of the model was verified by gender, multi-group analysis was used to identify significant differences in the model by sex (Marsh, 1987). Test results invariance (Byrne et al., 1989) showed that differences between groups in the model were not significant [*X*^2^ (122, *N* = 269) = 117.497; *p* = 0.598]. Therefore the three-wave longitudinal model is consistent and provides a robustness test for both sexes (Kong and Zhao, 2013).

## Discussion

This study was designed to examine the mediation of the relationships between PEI and life satisfaction by positive and negative affect in a sample of adolescents. Consistent with our hypothesis, correlational analysis showed that attention to feelings was positively correlated with negative affect (Augusto-Landa et al., 2006; Thompson et al., 2011), whereas mood clarity was positively correlated with positive affect (Gohm and Clore, 2002a; Palmer et al., 2002; Augusto-Landa et al., 2006; Rey et al., 2011) and life satisfaction (Palmer et al., 2002; Bastian et al., 2005; Extremera and Fernández-Berrocal, 2005). Emotional repair was positive correlated with positive affect (Gohm and Clore, 2002a; Augusto-Landa et al., 2006, 2012; Kafetsios and Zampetakis, 2008) and life satisfaction (Bastian et al., 2005; Extremera and Fernández-Berrocal, 2005; Augusto-Landa et al., 2006; Rey et al., 2011). Our data indicated that these association are stable over time and are consistent with previous studies showing that emotionally intelligent people can use positive emotions to manage anxiety and stress in the face of negative events (Tugade and Fredrickson, 2002) and that they are likely to use adaptive strategies, such as social support and emotional expression, rather than maladaptive strategies, such as rumination, to cope with stressful situations (Matthews et al., 2006). In general, it appears that emotional skill, as assessed by the TMMS dimensions, is negatively associated with physical symptoms of stress and positively associated with a more adaptive reaction to stressors. It may be that individuals who perceive their feelings clearly and believe that they can repair negative mood states turn their attentional resources toward coping with and minimizing the impact of stressful events (Salovey et al., 2002) whereas individuals with low scores on TMMS dimensions tend to engage in extended rumination in order to understand how they feel. Rumination and the absence of attempts to attend to, clarify, and repair mood might lead to prolonged physiological arousal and hence to negative health outcomes (Nolen-Hoeksema et al., 1994; Gross, 1998; Fernández-Abascal and Martín-Díaz, 2015). It is likely to retrieve positive memories as a way of regulating mood (Ciarrochi et al., 2000) and to take advantage of high social competence, extensive social networks, and effective coping strategies (Salovey et al., 2000). The individuals are able to identify and interpret emotional signals and regulate their actions autonomously, thus promoting positive affect and avoiding negative affect (Mayer and Salovey, 1997) and experiencing a sense of satisfaction with their lives. In line with these findings, our results provide evidence that adolescents with high PEI experienced more positive affect, less negative, and greater life satisfaction than those with lower PEI over the course of a 2-years period.

The most important finding of this study is that in adolescents positive and negative affect partially mediated the relationship between PEI and life satisfaction over a period of 2 years. The three-step longitudinal model of this study corroborates other reports that positive and negative affect act as mediators of the relationship between EI and life satisfaction (Gignac, 2006; Kong and Zhao, 2013), indicating that high PEI tends to results in more positive experiences and fewer negative experiences, thus contributing to greater life satisfaction. Kong and Zhao (2013) reported that the relationship between EI and life satisfaction was fully mediated by positive and negative affect, but we found only partial mediation, implying that PEI influences various life satisfaction factors indirectly by maintaining enabling individuals to maintain a positive affective balance. Our data are consistent with the model described by Gignac (2006). The differences between our finding and those of Kong and Zhao (2013) might be due to differences in the instruments used to measure PEI, the latent variables in the structural equation model or cultural differences between the samples.

This study also differs from previous studies in the use of TMMS dimensions to represent emotional variables in the model. Including the TMMS dimensions allowed us to assess how they were individually related to affective balance and life satisfaction. Attention to feelings was found to have more influence on negative affect than the component of PEI with positive affect, in line with previous results linking attention to feelings with negative ruminative processes over time. It has been suggested that rumination might be partly responsible for emotional distress and lack of well-being associated with greater attention to feelings (Fisher et al., 2010; Salguero et al., 2011). In general, our results support our initial hypothesis about the indirect effects of aspects of PEI on life satisfaction, providing perhaps the strongest evidence so far that PEI is an effective predictor of SWB.

This study was based on longitudinal data from a large sample of adolescents collected at three timepoints over a 2-years period. This longitudinal design allowed us to evaluate the timing of PEI and components of well-being. In line with our hypothesis, our results suggest that PEI plays a causal role in positive and negative affect and life satisfaction, and that affective balance is causally related to life satisfaction (Schimmack et al., 2008). Multi-group structural analysis confirmed that the temporal sequence model was identical for both sexes (Marsh, 1987), suggesting that the structure of the causal relationships is similar in both sexes.

Early research suggested that EI interventions improve well-being in young people (Brackett et al., 2010; Ruíz-Aranda et al., 2012), but the mechanisms underlying their effects were not understood. This study allowed us to evaluate how various emotional processes influenced SWB in adolescents (Qualter et al., 2012; Keefer et al., 2013). We found that all aspects of PEI influenced life satisfaction, positively in the case of mood clarity and emotional repair, and negatively in the case of attention to feelings. But an effect indirect predictors were also found through positive and negative affect (Gignac, 2006). This suggests that adolescents who pay less attention to their feelings experience less negative affect and more positive affect and hence feel greater satisfaction with their lives. Similarly, adolescents who perceived their mood more clearly and are better at emotional repair experience less negative affect and more positive affect and hence greater satisfaction with their lives (Frederickson et al., 2012; Gugliandolo et al., 2015). Becoming more aware one’s emotions and regulating them effectively are negatively associated with negative emotions (Salovey et al., 1999) and positively associated with positive emotions through time (Mikolajczak et al., 2008).

The findings of this study should, however, be interpreted in the light of several limitations. First, we did not assess personality traits, which are another potential influence on perceptions of well-being. Future research in this field should include some assessment of personality type (Andrei et al., 2014; Di Fabio and Saklofske, 2014). To increase generalisability of results future studies should use more diverse samples (Kristensen et al., 2014). We also recommend investigating whether these findings can be replicated in other cultures and nationalities (Bastian et al., 2014).

Despite these limitations the study makes an important contribution to understanding in this field and provides some support for a model of in which affective balance acts as a partial mediator of the relationship between EI and life satisfaction in adolescence. By using a longitudinal design we were able to probe the causal relationships between variables and achieve a better understanding of the chronology of the influence of PEI on affective and cognitive SWB. These findings could be used to improve interventions designed to increase adolescents’ sense of well-being.

## Conflict of Interest Statement

The authors declare that the research was conducted in the absence of any commercial or financial relationships that could be construed as a potential conflict of interest.
